# Pancreas transplantation improves the quality of life of Japanese type 1 diabetes patients with diabetic kidney disease

**DOI:** 10.20407/fmj.2022-019

**Published:** 2022-12-27

**Authors:** Chika Fujisawa-Tanaka, Izumi Hiratsuka, Megumi Shibata, Kei Kurihara, Naohiro Aida, Takeshi Takayanagi, Yusuke Seino, Taihei Ito, Takashi Kenmochi, Atsushi Suzuki

**Affiliations:** 1 Department of Endocrinology, Diabetes and Metabolism, Fujita Health University, School of Medicine, Toyoake, Aichi, Japan; 2 Department of Transplantation and Regenerative Medicine, Fujita Health University, School of Medicine, Toyoake, Aichi, Japan

**Keywords:** Pancreas transplantation, Quality of life, Type 1 diabetes mellitus, Diabetic kidney disease

## Abstract

**Objectives:**

Type 1 diabetes mellitus (T1DM) patients with diabetic kidney disease-induced kidney failure have a significantly impaired quality of life (QOL), resulting in a high level of physical, mental, and social anxiety. In this study, we evaluated the QOL of T1DM patients on the list for pancreas transplantation (PTx) at their registration, and determined whether PTx improved their QOL.

**Methods:**

There were 58 patients (men/women, 22/36; mean age, 42.8±8.0 years) with T1DM and who were registered on the waiting list for PTx. Quantitative QOL assessment was performed using the Medical Health Survey Short Form (SF-36) version 2. Changes in the QOL before and after PTx were also examined in 24 of these patients.

**Results:**

The mean value of each endpoint and the summary score of the SF-36 physical (PCS), mental (MCS), and role (RCS) components were all below the national normal level at PTx registration. No significant difference in QOL scores was observed in the intergroup comparison of 35 patients on dialysis, 13 patients without dialysis, and ten patients after kidney transplantation. The 24 patients who underwent PTx showed improvement in PCS, MCS, and most SF-36 scores.

**Conclusion:**

T1DM patients waiting for PTx had a decreased QOL, regardless of dialysis, and PTx improved their QOL.

## Introduction

Type 1 diabetes mellitus (T1DM) is a chronic disease caused by autoimmune isletitis, resulting in lifelong depletion of insulin secretion and a requirement for insulin therapy.^1^ Recent advances in insulin preparations and insulin delivery devices have greatly improved glycemic control in T1DM patients.^2^ However, it is still difficult to maintain normal blood glucose levels in T1DM patients, and diabetic complications, including diabetic kidney disease (DKD), result in kidney failure and reduce life expectancy and the quality of life (QOL).^3,4^

Pancreas transplantation (PTx) dramatically improves endogenous insulin secretion in T1DM patients, often eliminating the need for insulin injections.^5^ Recent advances in immunosuppressive therapy have improved the engraftment rate for pancreas transplantation. In Japan, the revised Organ Transplant Law has greatly increased the number of pancreas transplants from brain-dead donors, and 437 PTx were performed in Japan between 2000 and 2019.^6–8^ Simultaneous pancreas–kidney transplantation (SPK) could eliminate both insulin injections and maintenance dialysis and also improve life expectancy in T1DM patients with kidney failure.^9^ Since 2021, islet transplantation has been reimbursed by National Health Insurance in Japan, which should enable more T1DM patients to be offered the option of transplantation. T1DM patients waiting for PTx may have frequent hypoglycemia, which could disturb their daily life activities and might put their lives at risk. Despite progress in diabetes treatment, T1DM patients have a high prevalence of chronic complications, such as diabetic retinopathy, DKD, and neurological disorders. Patients with kidney failure due to DKD experience the burden of maintenance dialysis and the discomforts associated with dialysis, including dialysis disequilibrium syndrome.^10^ A low QOL for patients on the PTx waiting list and improvement in the QOL after PTx have been reported.^11^ Nyumura et al.^12^ reported that the post-transplant QOL of patients undergoing SPK was superior to that of patients on dialysis or those undergoing kidney transplantation (KTx) alone. However, KTx did not always improve the poor QOL in T1DM patients with kidney failure, and information about the effect of pre-transplant dialysis status on QOL and precise information on how PTx affects QOL after PTx are required. In this study, we investigated the QOL for T1DM patients before PTx at their enrolment in the PTx recipient registry and the impact of dialysis status on these patients’ QOL. Changes in the QOL before and after PTx were examined.

## Methods

### Patients

T1DM patients waiting for PTx were recruited at the Fujita Health University Hospital from 2011 to 2021 when they were registered on the waiting list for PTx. Fifty-eight patients (men/women, 22/36) agreed to participate in this study. Their mean age (±standard deviation, SD) was 42.8±8.0 years. There were 35 patients on chronic dialysis, 13 patients with DKD without dialysis, and ten post-KTx patients. Among the 58 patients, 24 had PTx, comprising 20 SPK and four pancreas transplantation after kidney transplantation (PAK), and we compared changes in the QOL score before and after PTx. Post-PTx questionnaires were completed once at least 1 year after PTx (maximum 3 years after PTx), from 2014 to 2021. This study was approved by the Review Board for Epidemiology and Clinical Studies of Fujita Health University (HM 21–433) and was conducted in accordance with the ethical standards of the 1964 Declaration of Helsinki and its later amendments. Written informed consent for the collection of clinical data including the questionnaire for the PTx registration was obtained from each participant, and the patients who did not wish to participate in this study had a chance to be excluded through an opt-out system.

### QOL questionnaire

To evaluate the QOL, we used the Medical Outcome Health Survey Short Form 36 (SF-36) version 2.^13,14^ SF-36 is a self-administered survey that is a nonspecific method of evaluating the overall QOL. SF-36 consists of eight subscales, the scores of which are entered into the scoring program and normalized in accordance with the 2007 reference values for the Japanese population to obtain a mean of 50 points and a SD of 10 points. The SF-36 subscales are physical functioning (PF), role-physical functioning (RP), body pain (BP), general health (GH), vitality (VT), social functioning (SF), role-emotional functioning (RE), and mental health (MH). Scores from these eight subscales were then used to calculate the following three summary scores: the physical component summary score (PCS), the mental component summary score (MCS), and the role/social component summary score (RCS). The reliability of the Japanese version of the SF-36 version 2 has been validated.^13,14^

### Biochemical measurements

Clinical information and laboratory data were obtained from each patient using plasma or serum when the patients were registered on the waiting list for PTx. Glycated hemoglobin (HbA1c), C-peptide immunoreactivity (CPR), hemoglobin (Hb), estimated glomerular filtration rate (eGFR), and creatinine (Cr) were measured using automated techniques in the central laboratory at this hospital (Adams A1c HA8181 and HA8190V of Arkray Inc., Kyoto, Japan; LABOSPECT 008 of Hitachi High-Tech Co., Tokyo, Japan). The acute response to glucagon was measured by an increase in CPR levels from 0 to 6 minutes after a 1-mg glucagon challenge (ΔCPR).^15^ Serious hypoglycemia during the last year and unconscious hypoglycemia were defined on the basis of self-reporting and data from continuous glucose monitoring at registration. Hypertension was defined using the diagnostic criteria for hypertension from the Japanese Society of Hypertension.^16^ Retinopathy was identified through examination by expert ophthalmologists. Neuropathy was identified by examining the diagnostic criteria for diabetic polyneuropathy from the Japanese Study Group of Diabetic Neuropathy.^17^ Peripheral artery disease (PAD) was defined using the ankle brachial pressure index (ABI). Ischemic heart disease (IHD) and stroke were determined from the patient’s medical history.

### Statistical analysis

Continuous variables are expressed as the arithmetic mean±SD or as the median value (interquartile range). Between-group comparisons of continuous variables in two groups were conducted using a paired *t*-test. Comparisons of continuous variables among three groups were conducted using an analysis of variance. Additionally, p values less than 0.05 were considered to be statistically significant. All analyses were performed using the Stat Flex Ver 7.0.11 (Artech Co, Ltd, Osaka, Japan).

## Results

### QOL evaluation of T1DM patients at PTx registration

Tables 1 and 2 show the clinical backgrounds of 58 T1DM patients (men/women, 22/36), including 35 patients on dialysis (mean duration on dialysis, 3.7±4.8 years), 13 patients without dialysis (mean eGFR, 44.8±39.3 mL/min/1.73 m^2^), and ten post-KTx patients (mean eGFR, 43.0±25.1 mL/min/1.73 m^2^). All patients underwent insulin therapy (mean insulin dose, 29.7±13.9 units per day), and their mean HbA1c levels were 7.4±1.4%. Their insulin secretion was almost completely inhibited (mean ΔCPR, 0.02±0.09 ng/mL).

**Figure 1:**
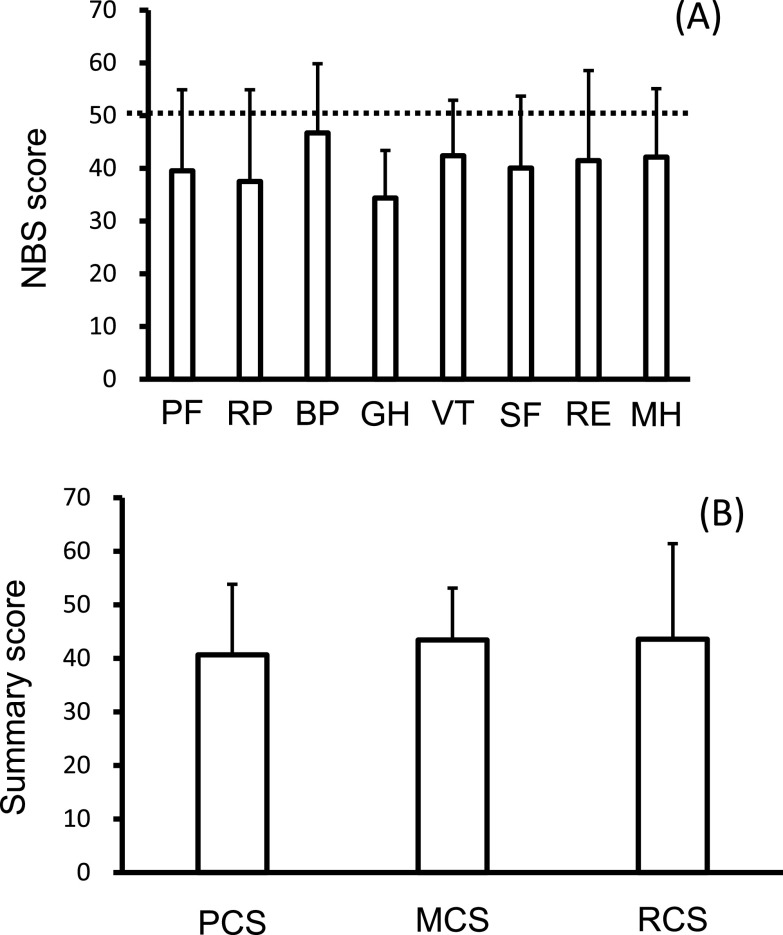
NBS50 (A) and summary scores (B) of the SF-36 at registration for pancreas transplantation. Data are presented as the mean±SD. PF, physical functioning; RP, role physical; BP, bodily pain; GH, general health; VT, vitality; SF, social functioning; RE, role emotional; MH, mental health; PCS, physical component summary; MCS, mental component summary; RCS, role component summary; SD, standard deviation; NBS50, norm-based scoring 50; SF-36, Medical Health Survey Short Form

**Figure 2:**
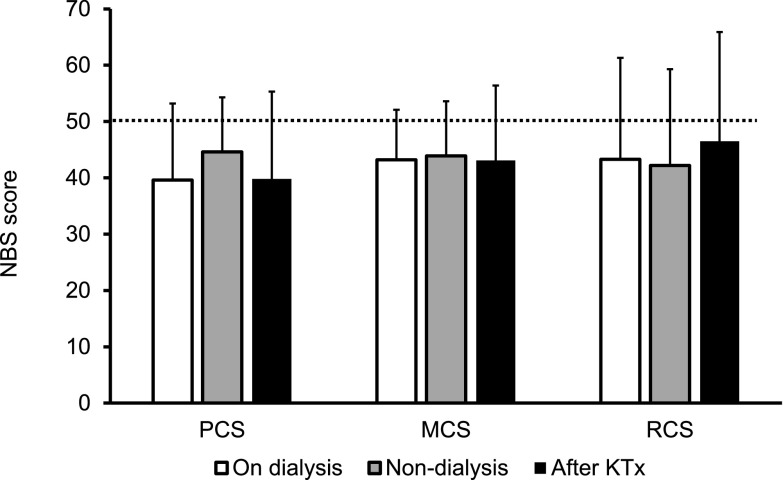
Norm-based summary scores on the basis of the dialysis status at the time of registration for pancreas transplantation. Data are presented as the mean±SD. KTx, kidney transplantation; PCS, physical component summary; MCS, mental component summary; RCS, role component summary; SD, standard deviation

Diabetic microvascular complications were common at registration. There were 53 diabetic neuropathy patients and 51 diabetic retinopathy patients, including four patients who were blind and 51 patients with diabetic nephropathy. Figure 1A shows the SF-36 norm-based scoring (NBS) scores at PTx registration; the mean values of each SF-36 endpoint component were all below the national normal level. The scores were as follows: PF, 39.5±15.4; RP, 37.5±17.4; BP, 46.7±13.1; GH, 34.4±9.0; VT, 42.4±10.5; SF, 40.0±13.7; RE, 41.4±17.1; and MH, 42.2±12.9. The mean SF-36 summary score values such as PCS (40.6±13.2), MCS (43.4±9.7), and RCS (43.6±17.8) were all below the national normal level at PT registration (Figure 1B). Next, we analyzed the SF-36 summary scores on the basis of dialysis status. There was no difference in the QOL score (Figure 2) or incidence of serious hypoglycemia among the groups (on dialysis: Yes/No, 28/7; non-dialysis: Yes/No, 10/3; after KTx: Yes/No, 8/2).

### Comparison of QOL scores before and after PTx

QOL scores before and after PTx were compared in 24 patients, including 20 SPK and four PAK patients (Table 3). The mean ages before and after PTx were 41.7±8.7 years and 45.5±8.6 years, respectively. There were 16 patients on dialysis (mean duration of dialysis, 3.3±3.6 years), five post-KTx patients, and three non-dialysis patients. The average insulin use pre-PTx was 27.8±12.3 units per day. The insulin secretory capacity recovered (ΔCPR, 3.84±2.30 ng/mL) after PTx, and all post-PTx patients became insulin-free. Their mean pre-PTx HbA1c level was 7.0±0.9%, and it improved to 5.2±0.4% after PTx.

**Figure 3:**
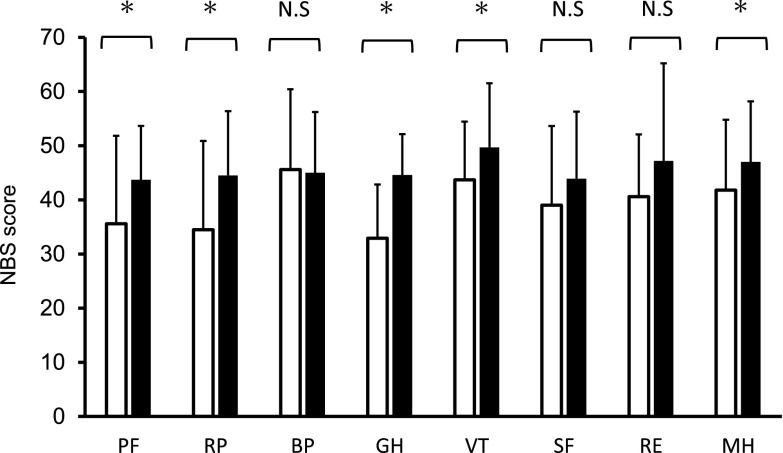
NBS50 before (open bars) and after (closed bars) pancreas transplantation. Data are presented as the mean±SD. * p<0.05 N.S., not significant; NBS, norm-based scoring; NBS50, Norm-based scoring 50; PF, physical functioning; RP, role physical; BP, bodily pain; GH, general health; VT, vitality; SF, social functioning; RE, role emotional; MH, mental health; SD, standard deviation

**Figure 4:**
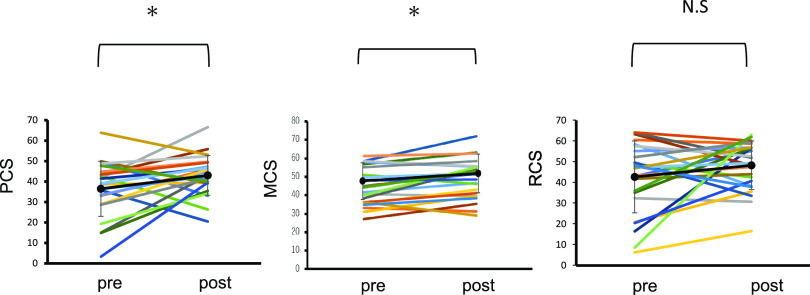
Norm-based summary scores in each patient before and after pancreas transplantation. Data are presented as the mean±SD. * p<0.05 N.S, not significant; PCS, physical component summary; MCS, mental component summary; RCS, role component summary; SD, standard deviation

PTx improved the NBS score for five items in the SF-36 version 2.0 (Figure 3). Each score was as follows: PF (pre/post), 35.6±16.2 / 43.7±10.0 (p<0.01); RP (pre/post), 34.5±16.4 / 44.5±11.9 (p=0.01); BP (pre/post), 45.6±14.8 / 45.0±11.2 (p=0.85); GH (pre/post), 32.9±9.9 / 44.6±7.6 (p<0.01); VT (pre/post), 43.7±11.8 / 49.7±10.7 (p=0.02); SF (pre/post), 39.0±14.6 / 43.9±12.4 (p=0.18); RE (pre/post), 40.6±18.0 / 47.2±11.5 (p=0.08); and MH (pre/post)=41.8±13.0 / 47.0±11.2 (p=0.02). Summary scores in PCS (pre/post) were 36.8±13.4 / 43.2±9.6 (p=0.03), and that for MCS (pre/post) was 44.8±9.9 / 49.0±10.5 (p<0.01) (Figure 4). The scores improved significantly after PTx, but there was no significant change in RCS after PTx (pre/post=42.1±17.3 / 48.2±11.7 (p=0.14).

## Discussion

In this study, we found that T1DM patients waiting for PTx had low QOL scores, regardless of their hemodialysis status. Additionally, most QOL scores improved after PTx, suggesting that PTx could contribute to a better QOL in T1DM patients with DKD.

T1DM patients are more likely to become insulin-dependent than those with type 2 diabetes and require life-long insulin self-injection to maintain their blood glucose levels.^1^ T1DM patients have impairment in both insulin and glucagon secretion, resulting in wide fluctuations of blood glucose levels in their daily lives. Generally, blood glucose control in T1DM is more difficult compared with that in T2DM. Accumulated glycation end-products, resulting from high blood glucose levels, can contribute to diabetic complications, such as diabetic retinopathy, neuropathy, and DKD. Among these, DKD caused a decline in renal function and induced kidney failure.^4^ Patients with kidney failure eventually need persistent hemodialysis, which limits their daily life activities. In this study, we found that T1DM patients waiting for PTx had low QOL scores, regardless of their dialysis status. Although KTx for hemodialysis patients could contribute a better QOL,^3,18^ post-KTx T1DM patients were reported to have the same SF-36 summary scores as T1DM patients on hemodialysis.^12^ We showed here that PTx (mainly SPK) improved most QOL scores in our patients. Another group also reported that SPK recipients had higher SF-36 summary scores than patients remaining on the waiting list for PTx.^19^ These results suggest that hemodialysis is unlikely to be the main factor that reduces the QOL of T1DM patients with kidney failure and that KTx alone might be insufficient to improve the QOL in these patients. SPK can free T1DM patients from persistent dialysis and insulin injection. Thus, PTx could eliminate the fear of life-threatening hypoglycemia for these patients. In this study, T1DM patients with kidney failure and who are waiting for PTx had a high incidence of serious hypoglycemia, regardless of their dialysis status. Hypoglycemia was eliminated in all the PTx recipients after PTx. Except for relief from hemodialysis and insulin injection, SPK also improved other diabetic complications, such as diabetic neuropathy.^20^ We reported previously that serious atonic gastroenteropathy and orthostatic hypotension improved after SPK.^21^ PTx may also contribute to improving eye conditions.^22^

After PTx, physical health components such as PF and RP improved, and the patients had better GH and PCS compared with those before PTx. PF consists of physical activities such as walking, stair climbing, and lifting luggage in daily life, and RP comprises the quality and quantity of daily activities. Therefore, the improvement in physical ability due to PTx should contribute to the patient’s general physical health condition.

Psychological symptoms are prevalent in pre-SPK and post-SPK patients, which could reduce the QOL of T1DM patients with kidney failure.^23^ In this study, we found that SPK improved VT, MH, and MCS in post-PTx patients. In Western countries, post-PTx patients had higher PCS but not MCS because there was less psychological distress from diabetes.^18,24^ Longer waiting periods for SPK in Japan may affect mental function because longer waiting periods could cause mental health to deteriorate in T1DM patients.^12^ Therefore, the pre-PTx mental condition might eliminate pre-PTx MCS, and the difference between pre- and post-PTx could be more apparent in our study compared with that in previous reports.

However, we found here that improvements in SF, RE, and RCS post-PTx were not significant. T1DM onset typically occurs most often in young children and teenagers, and these patients need long-term treatment with insulin during adolescence.^1^ Both severe and non-severe hypoglycemic episodes contribute to the economic and psychological burdens.^25^ Additionally, patients with kidney failure had a low employment rate both during ongoing hemodialysis and after KTx.^26^ Socioeconomic disparities in T1DM patients are likely to lead to worse outcomes, such as high mortality and morbidity.^27,28^ Even after PTx, including SPK, socio-economic issues could be a burden for T1DM patients, and continuous support is necessary for post-PTx patients.

There are some limitations to this study. First, it was conducted at a single center. Second, the period for assessing the patients’ post-PTx QOL was 1–3 years after PTx, and a longer follow-up period would provide more information about the QOL with other diabetic complications. Third, the number of PAK patients was small, and we could not compare the impact of PTx with that of KTx on T1DM patients with kidney failure.

In conclusion, T1DM patients waiting for PTx had a decreased QOL, regardless of dialysis, and PTx improved their QOL.

## Figures and Tables

**Table1:** Clinical characteristics of patients with type 1 diabetes waiting for pancreas transplantation at the time of registration for pancreas transplantation

	N=58
Age (years)	42.8±8.0
Sex (Men:Women)	22:36
Age of onset (years)	16.7±8.5
Duration dialysis (years)	3.7±4.8 (N=35) (Non-dialysis: N=13/After KTx: N=10)
Insulin dosage (U/day)	29.7±13.9
HbA1c (%)	7.4±1.4
ΔCPR (ng/mL)	0.02±0.09
Hb (g/dL)	11.0±1.7
eGFR (mL/min/1.73 m^2^)	44.0±32.9 (Non-dialysis: N=13/After KTx: N=10)
Cr (mg/dL)	6.2±4.0

Data are presented as the mean±SD. KTx, kidney transplantation; HbA1c, hemoglobin A1c; ΔCPR, the elevation of C-peptide immunoreactivity level from 0 to 6 min after 1 mg glucagon challenge; Hb, hemoglobin; eGFR, estimated glomerular filtration rate; Cr, creatinine; SD, standard deviation

**Table2:** Complications in patients with type 1 diabetes waiting for pancreas transplantation at the time of registration for pancreas transplantation

		N=58
Serious hypoglycemia in the last year	Yes	46/58
	No	12/58

Unconscious hypoglycemia	Yes	54/58
	No	1/58
	Uncertain	3/58

Hypertension		40/58

Retinopathy	NDR	7/58
	SDR	3/58
	PPDR	6/58
	PDR	38/58
	Blind	4/58

eGFR (mL/min/1.73 m^2^)	Non-dialysis (N=13)	44.8±39.3
	After KTx (N=10)	43.0±25.1
	On dialysis (N=35)	n.a.

Neuropathy		53/58
PAD		8/58
IHD		0/58
Stroke		1/58

Data are presented as the mean±SD. NDR, no diabetic retinopathy; SDR, simple diabetic retinopathy; PPDR, pre-proliferative diabetic retinopathy; PDR, proliferative diabetic retinopathy; KTx, kidney transplantation; PAD, peripheral artery disease; IHD, ischemic heart disease; SD, standard deviation; n.a., not applicable

**Table3:** Analysis of patients’ clinical characteristics on the basis of the change in SF-36 scores before and after pancreas transplantation

N=24	Pre-PTx		Post-PTx (SPK=20, PAK=4, PTA=0)
Age (years)	41.7±8.7		45.5±8.6

Sex (Men:Women)	8:16		

HD	On dialysis	N=16 3.3±3.6 years	After KTx N=24
	After KTx	N=5	
	Non-dialysis	N=3	

Insulin dose (U/day)	27.8±12.3		0

Serious hypoglycemia	Yes=22 No=0 Uncertain=2		Yes=0 No=24

ΔCPR (ng/mL)	0.002±0.006		3.84±2.30

HbA1c (%)	7.0±0.9		5.2±0.4

eGFR (mL/min/1.73 m^2^)	40.1±29.8 (N=8)		50.7±14.1

Cr (mg/dL)	6.60±3.77		1.11±0.28

Data are presented as the mean±SD. SPK, simultaneous pancreas and kidney transplantation; PAK, pancreas transplantation after kidney transplantation; PTA, pancreas transplantation alone; HD, hemodialysis; ΔCPR, C-peptide immunoreactivity; HbA1c, hemoglobin A1c; eGFR, estimated glomerular filtration rate; Cr, creatinine; KTx: kidney transplantation; SD, standard deviation
